# Continuous Glucose Monitoring in Enterally Fed Children with Severe Central Nervous System Impairment

**DOI:** 10.3390/nu15030513

**Published:** 2023-01-18

**Authors:** Marlena Górska, Joanna Kudzin, Anna Borkowska, Agnieszka Szlagatys-Sidorkiewicz, Agnieszka Szadkowska, Małgorzata Myśliwiec, Ewa Toporowska-Kowalska

**Affiliations:** 1 Department of Pediatric Allergology, Gastroenterology and Nutrition, Medical University of Lodz, 90-419 Lodz, Poland; 2 Department of Pediatrics, Gastroenterology, Allergology and Nutrition, Medical University of Gdańsk, 80-210 Gdańsk, Poland; 3 Department of Pediatrics, Diabetology, Endocrinology and Nephrology, Medical University of Lodz, 90-419 Lodz, Poland; 4 Department of Pediatrics, Diabetology and Endocrinology, Medical University of Gdańsk, 80-210 Gdańsk, Poland

**Keywords:** enteral nutrition, tube feeding, gastrostomy, neurological impairment, continuous glucose monitoring, hypoglycaemia

## Abstract

Children with severe central nervous system (CNS) impairment are at risk of developing various degrees of nutritional deficit that require long-term nutritional intervention. Interventions are most often implemented through enteral nutrition (EN) using commercially manufactured feeds administered via gastro/jejunostomy or nasogastric or nasojejunal tubes. The modality of feeding—continuous feeding or bolus feeding—is dependent on the function of the gastrointestinal tract, particularly the efficiency of gastric emptying. In the literature, the relationship between this type of nutrition and the occurrence of hyperglycaemia is often discussed. In addition, children with chronic neurological diseases are vulnerable to disorders of many mechanisms of neurohormonal counter-regulation related to carbohydrate management, and due to limited verbal and logical contact, it is difficult to recognise the symptoms of hypoglycaemia in such patients. We aimed to assess the carbohydrate metabolism in children with severe CNS impairment, with enteral nutrition delivered via nasogastric, nasoenteral, or percutaneous tubes, based on continuous glycaemic monitoring (CGM) and the measurement of glycated haemoglobin (HbA1c) levels. Materials and methods: This prospective, observational study included nineteen patients (median (25–75 pc) age: 12.75 (6.17–15.55) years) with permanent CNS damage (Gross Motor Function Classification System V) receiving long-term tube enteral feeding, recruited from two paediatric university nutritional treatment centres. Patients with acute conditions and diagnosed diabetes were excluded. The nutritional status and nutritional support were analysed in all the inpatients in accordance with a uniform protocol. Using the CGM system (Medtronic iPro2), glycaemic curves were analysed, and in addition, HbA1C levels were determined in fourteen patients. CGM results were analysed using GlyCulator2.0. Statistical analysis was performed using the Statistica version 11 software (StatSoft Inc. Tulsa, OK, USA). Results: More than half (11/19; 58%) of the patients were undernourished (BMI < 3 pc for age and gender), with the stature age being significantly lower than calendar age (5 (4.5–9) vs. 12.75 (6.17–15.55) years; *p* = 0.0010). The actual caloric intake was 50 (37.7–68.8) kcal/kg (median; 25–75 pc). In patients fed using the bolus method, the number of calories consumed per day was statistically significantly higher than in children subjected to a continuous feeding supply (56.00 (41.00–75.00) vs. 33.40 (26.70–50.00) kcal/kg BW (body weight; *p* = 0.0159). Decreases in blood glucose levels below the alarm level (<70 mg/dL) were recorded in fifteen patients (78.9%), including two patients with episodes of clinically significant hypoglycaemia (<54 mg/dL). The minimum and maximum glycaemic values recorded in any individual CGM records were 67 mg/dL (median) (minimum: 41 mg/dL; maximum: 77 mg/dL) and 146 (minimum: 114 mg/dL; maximum: 180 g/dL), respectively, for the entire recording. The maximum percentage of glycaemic concentrations > 140 mg/dL (TAR 140) recorded overnight in children with BMI ≥ 3 amounted to 1.6% vs. 0% in undernourished patients (TAR 140: 0.0 (0.00–1.6%) vs. 0% (0.00–0.0%; *p* = 0.0375); the percentage of glycaemic concentrations <70 mg/dL in the entire recording was comparable (0.77% (0.13–2.2%) vs. 1.8% (0.5–14.4%) vs. *p* = 0.2629). There was a positive correlation between the mean daily glucose recorded using the CGM method and patients’ BMI z-scores (R = 0.48, *p* = 0.0397). No statistically significant relationship was demonstrated between the occurrence of alarm hypoglycaemia events in the CGM records and undernutrition expressed by BMI z-scores (OR = 1.50 (95%CI: 0.16–13.75), the type of diet (for commercially manufactured OR = 0.36 (95%CI: 0.04–3.52), and the modality of diet delivery (for bolus feeding OR = 2.75 (95%CI: 0.28–26.61). Conclusions: In children with chronic OU damage, enteral feeding is associated with a risk of hypoglycaemia, but further studies involving a larger number of patients are needed, and CGM might be a useful tool to estimate the metabolic adequacy of enteral nutritional support in terms of glucose control.

## 1. Introduction

Children with severe impairments of the central nervous system (CNS) are at risk of developing malnutrition and growth failure, the treatment of which requires long-term nutritional support, most often implemented through enteral nutrition (EN) using standardised commercially prepared formulas administered via gastrostomy, gastro-jejunostomy, nasogastric (NG), or nasojejunal (NJ) tubes [1,2]. Equations (the Schofield or Harris-Benedict formulas), including coefficients depending on the level of physical activity and the degree of undernutrition, are used to determine energy requirements [3]. In clinical practice, however, the volume of the feed provided is limited by individual patients’ tolerance, primarily depending on the motor performance of the gastrointestinal tract [4]. Monitoring the effects and safety of a nutritional treatment is based on the assessment of anthropometric parameters, body composition, and biochemical measurements [1,2,5]. One of the primary indicators of systemic homeostasis is glucose concentration, which in children fed enterally is routinely assessed via single point-of-care serum/capillary blood measurements [1]. Significant advancements in the control of glycaemic disorders have been brought into clinical practice through continuous glucose monitoring (CGM), which is primarily used to assess metabolic control in patients with diabetes [6]. Isolated publications which describe daily glycaemic profiles in non-diabetic enterally fed patients, e.g., neonates or ICU patients, are available, but no studies have been carried out regarding children with CNS impairment [7].

In this study, we aimed to assess the carbohydrate metabolism in children with severe CNS impairment who received enteral nutrition delivered via nasogastric, nasoenteral, or percutaneous tubes, based on continuous glycaemic monitoring (CGM) and the measurement of glycated haemoglobin (HbA1c) levels.

## 2. Materials and Methods

This prospective study was carried out as part of a multi-centre project which aimed to monitor the effectiveness and complications of enteral nutrition in children with central nervous system impairment and included patients under the constant care of two university-based paediatric nutritional treatment centres. The study was conducted in a hospital inpatient setting.

### 2.1. Subjects

A total of 19 children (10 boys and 9 girls) with CNS impairment were enrolled in the study. All the patients met the following inclusion and exclusion criteria: 

Inclusion criteria were: severe CNS impairment requiring enteral nutrition delivered via feeding tubes (gastrostomy/gastro-jejunostomy or nasogastric/nasojejunal tubes); age between 3 and 18 years old; enteral nutrition used for at least 6 months; stable phase of nutritional treatment—target/maximum tolerated volume of feed achieved; and informed consent from parents/legal guardians.

Exclusion criteria included: the occurrence of factors that may affect glucose results (current infection, fresh trauma, the introduction of new drugs (including glucocorticoids)), previously diagnosed diabetes; no informed consent from legal guardian; and patients ≥ 16 years of age.

In thirteen out of nineteen (68.4%) children, cerebral palsy was diagnosed, with the remaining six (31.6%) being classified as having encephalopathies of another origin (including one patient with congenital CNS malformation). 

The median (25–75 pc) age of the patients was 12.75 (6.17–15.55) years. Children with cerebral palsy were older than the patients with other diagnoses (15.50 (12.75–16.42) vs. 5.17 (3.67–6.17) years; *p* = 0.0010).

The group was homogeneous in terms of the degree of motor dysfunction; all patients had disorders corresponding to GMFCS Level V according to the Gross Motor Function Classification System [8,9], and all patients presented with severe intellectual disabilities.

### 2.2. Nutritional Status Assessment

According to a uniform protocol, the subjects’ nutritional status parameters—body weight to the nearest 0.1 kg and linear growth indicators (body length/height/segmental lengths—ulnar or tibial length, using mathematical formulas to estimate height according to clinical status)—were assessed [10]. A growth age (the age for which the 50th percentile of the height standard corresponds to the current height/length of the child) was calculated for each subject [11]. The BMI, weight, and length for age z-scores were calculated based on anthropometric measurements. For the analysis, the WHO centile grids were used in the group of children up to 5 years of age, and the current national grids for the population of healthy children were used for patients aged 6–18 years old [12,13]. The threshold for undernutrition was defined as BMI ≤ 3 pc, >85 pc for overweight, and >90 pc for obesity [14]. 

### 2.3. Nutritional Support Assessment

In each patient, the route of enteral feeding (intragastric or postpyloric jejunal), mode of enteral access (percutaneous endoscopic gastrostomy—PEG; percutaneous endoscopic gastro-jejunostomy—PEG-PEJ; nasogastric tube—NG; nasojejunal tube—NJ), modality of diet delivery (continuous feeding with the use of enteral pump—CF; bolus feeding—BF) and type, the amount and composition of the diet (standard commercial formula—SCF; blenderised home-prepared diet—HD) were assessed. The actual energy intake (ACI) was estimated, and the values obtained were compared to the recommended daily caloric intake (RCI) calculated for each patient according to the Schofield formula with appropriate coefficients, considering the level of physical activity and undernutrition [1,15]. 

### 2.4. Carbohydrate Metabolism Assessment

Carbohydrate metabolism was assessed with the continuous glucose monitoring system and glycated haemoglobin (HbA1c) concentration measurement. Continuous glucose monitoring (CGM) was performed using a prospective, blinded method, using a sensor placed in the subcutaneous tissue on the abdomen for a minimum of 36 h (iPro, Medtronic, Minneapolis, MN, USA). At least three times a day, capillary blood glucose levels were measured using a glucometer (Accucheck Performa, Roche Diabetes Care), which was necessary to calibrate the CGM system, and the times of enteral feeding were recorded. Once the recordings were completed, the electrodes were removed, and data from the recording devices were exported and analysed using the GlyCulator 2.0 application [16]. The following parameters were assessed: the duration of CGM; the number of measurements recorded per patient; the mean, minimum, and maximum glucose levels; the number of hypoglycaemic events (<70 mg/dL and <54 mg/dL), including nocturnal hypoglycaemias; the number of hyperglycaemic events (>140 mg/dL); time in the range of 70–140 mg/dL (TIR); time above the range (>140 mg/dL—TAR); time under the range of <70 (TUR 70); time under the range of <54 mg/dL (TUR 54); and the coefficient of variation (CV) [17].

The concentrations of HbA1c were determined using the High-Performance Liquid Chromatography (HPLC) method (Bio-Rad Variant, Bio-Rad Laboratories, Hercules, CA, USA). The range of normal values was 4.8% to 5.7% [18].

### 2.5. Statistical Analysis

Statistical analysis was performed using Statistica version 11 (StatSoft Inc.Tulsa, OK, USA). The data were presented as a mean, along with a standard deviation and a median with quartile ranges. For variables with distributions that differed from the norm, the Mann–Whitney U non-parametric test was used. Spearman’s rank correlation coefficient was used to describe the strength of the correlations. The level of statistical significance was assumed to be *p* ≤ 0.05.

## 3. Results

### 3.1. Nutritional Status and Nutritional Support

Analyses of the nutritional status and nutritional support of the children included in the study are presented in Table 1. 

More than half (11/19; 58%) of the patients were undernourished (BMI < 3 pc for age and gender), and one child was obese (5.26%). Thirteen of the patients received a normocaloric (1 kcal/mL), and six received a hypercaloric (1.5 kcal.mL), polymeric diet.

The actual and recommended caloric intakes of patients in this study are shown in Table 2.

In patients fed using the bolus method, the number of calories consumed per day was statistically significantly higher than in children subjected to the provision of continuous feeding (56.00 (41.00–75.00) vs. 33.40 (26.70–50.00) kcal/kg BW; *p* = 0.0159). 

It was observed that children with cerebral palsy tolerated lower numbers of calories than patients with encephalopathy of other origins (41.00 (37.20–55.00) vs. 66.15 (56.00–90.90) kcal/kg BW; *p* = 0.0253). 

### 3.2. Carbohydrate Metabolism 

The parameters that were obtained in the CGM are presented in Table 3.

**Figure 1:**
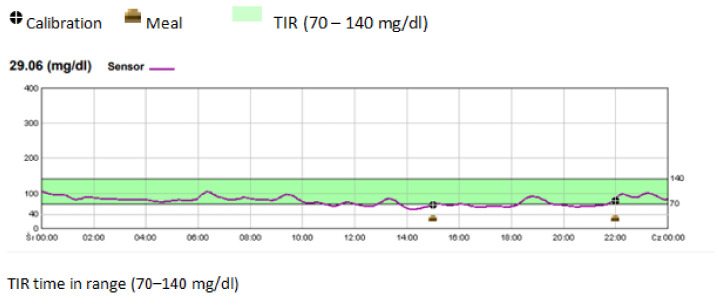
Continous glucose monitoring - example of severe hypoglycemia episodes in gastrostomy fed patient.

Drops in blood glucose below the level of 54 mg/dL were recorded in two patients (one and three episodes per patient, respectively); their clinical data are presented in Table 4. The 24 h CGM report displaying hypoglycaemia episodes in a patient receiving continuous feeding via a gastrostomy tube is presented in Figure 1.

The clinical characteristics and CGM parameters of the patients included in this study with respect to BMI-pc are shown in Table 5.

There was a positive correlation between the mean daily glucose concentrations recorded using CGM and the BMI z-scores of the patients (R = 0.48, *p* = 0.0397). 

No statistically significant relationship was demonstrated between the occurrence of alarm hypoglycaemia events in the CGM records and undernutrition expressed in the BMI z-scores (OR = 1.50 (95%CI: 0.16–13.75)), the type of diet (for commercially manufactured diets, OR = 0.36 (95%CI: 0.04–3.52)), and the modality of diet delivery (for bolus feeding, OR = 2.75 (95%CI: 0.28–26.61)).

Of the 14 patients whose HbA1C levels were measured, the results from five were below the recommended standard (35.7%). No patients showed elevated HbA1c levels.

## 4. Discussion

Enteral nutrition has become a standard procedure in the care of patients with severe CNS impairment and, in many cases, can be considered a life-saving medical intervention.

Children with chronic neurological disabilities of various aetiologies constitute the dominant group in the paediatric home enteral nutrition (HEN) registries kept in European countries (about 65% in Italy, 30.5% in Spain, 35% in France, and 30% in the United Kingdom); research shows that malnutrition affects as many as 46%–90% of patients with cerebral palsy [19,20,21]. 

The more severe the neurological impairment, the more severe the degree of undernutrition is [22]. The aetiology of undernutrition in patients with severe CNS impairments is multifactorial, resulting from disorders of gross and fine motor skills, impaired cognitive functions, and the inability to communicate hunger and satiety. Fine motor skills disorders are expressed by difficulties ingesting and chewing as well as oropharyngeal dysphagia (OPD); swallowing disorders may also be the result of oesophageal strictures complicating GORD. Dysphagia affects almost all patients with severe motor disorders (GMFCS grades IV and V); for example, in a study that included 1357 paediatric patients with cerebral palsy, Calis et al. reported moderate to severe dysphagia in 76% of patients, severe dysphagia in 15% of patients, and mild dysphagia in 8% of patients [23]. The overall incidence rate of swallowing disorders was 43%. The ESPGHAN (the European Society for Paediatric Gastroenterology, Hepatology and Nutrition) recommends the assessment of swallowing performance in all children with CNS impairment [1]. 

The recommended treatment for undernutrition in children with severe CNS impairment is enteral nutrition, most commonly realised with a gastrostomy; in clinically justified situations, long-term nasogastric/nasojejunal tube feeding is accepted [1,2]. Intragastric feeding is the preferred route of enteral diet delivery, and it was applied in seventeen (89,5%) of our patients, in all but one, in sixteen (84%), via percutaneous endoscopic gastrostomy. In the case of intolerance towards intragastric feeding and in patients for whom it is difficult to achieve the required nutritional intake, postpyloric access is advised, which was used in two children (the PEG-PEJ method). The use of standardised ready-to-use formulae is advocated, albeit some patients are fed a mixed diet or solely a homemade blenderised diet (as determined by parental/guardian preference). It should be emphasised that as the use of so-called blenderised diets in tube-fed patients arouses increasing levels of interest, the assessment of its nutritional value, safety, and cost-effectiveness remains ambiguous [24]. Recent data suggest that the utilisation of real food containing enteral formulas can increase caloric intake, support intestinal motility, and promote adequate weight-for-height gain in patients receiving long-term tube feeding [25]. Among the patients participating in our study, in 13 (68.4%), the exclusive standardised commercial diet was administered, and in 6 (31.6%), a mixed diet, including blenderised home-prepared feeds, was administered. In the case of a mixed diet, at least 50% of the RCI came from the standardised commercial formula, which is related to the HEN reimbursement rules set by the payer (the National Health Fund). 

Depending on the motoric efficiency of the gastrointestinal tract, enteral diets are administered as bolus or continuous feeding. In our study, in the majority of children (13 or 68%), boluses were administered. In this group, the number of calories consumed per day was statistically significantly higher than that in children subjected to the continuous feeding method (56.00 vs. 33.40 kcal/kg BW; *p* = 0.0159), which reflects the better general and digestive tract function of bolus-tolerant children rather than the efficiency of the feeding method.

Despite the long-term nutritional support provided (tube feeding with a maximum tolerated caloric intake for at least 6 months), patients presented a significant degree of undernutrition with a median BMI z-score of −2.26 (−4.41–(−1.07)) and a stature age substantially lower than metric one. The obtained parameters, in the absence of dedicated percentile grids for children with severe CNS impairment, were referred to general population standards in accordance with the NASPGHAN (North American Society for Paediatric Gastroenterology, Hepatology, and Nutrition) guidelines [22]. However, it should be noted that patients with extreme disabilities who are completely immobile differ significantly from healthy children in terms of body composition, which concerns the proportion of lean and fat mass and may affect the interpretation of the anthropometric measurements obtained. The more precise qualitative assessment of the nutritional status would therefore require the application of additional methods, such as skinfold and MUAC (mid-upper arm circumference) measurements, laboratory parameters, and DXA or bioimpedance procedures, which were not employed in our pilot study.

The CGM carried out in the study group is, to the best of our knowledge, the first attempt to use this method to assess the carbohydrate metabolism in enterally fed children with central nervous system impairment. Originally, the system was developed to improve glycaemic control in patients with diabetes, and also, the standards for interpreting the CGM readings are dedicated to this group of subjects [26]. Subsequently, the method was deployed in other clinical situations related and not related to disordered glucose metabolism (e.g., hypoglycaemia in neonates, patients in critical conditions, sport medicine, and healthy individuals) [27]. We have shown that for patients with chronic CNS damage who are tube-fed, hypoglycaemia can be a significant clinical problem. In our patients, the median TUR 70 amounted to 1.32 (0.39–3.03)%, and hypoglycaemia alarm events were detected in as many as 15 out of the 19 patients (79%), including two who experienced clinically significant decreases in glucose concentrations (TUR 54: 11% and 2.74%, respectively; Table 3) In healthy subjects, the percentage of time spent with glycaemia <70 mg% was estimated to be at the level of 0.4% (corresponding to 6 min/day) in children 1–6 years old and 1.1% (15 min/day) in the older population (7–80 years); at least one episode of hypoglycaemia occurred in 23% and 28% of the subjects in these groups, respectively [28,29].

The clinical picture of hypoglycaemia consists of symptoms of autonomic stimulation (e.g., sweating, palpitations, anxiety, hunger, paraesthesia, and hypothermia) and neuroglycopenia (e.g., drowsiness, confusion, and seizures) [30]. In our patients presenting with severe CNS impairment, hypoglycaemia was diagnosed on the basis of defined CGM criteria (as at least two sensor values <54 mg (<70 mg/dL) that were ≥15 min apart with no intervening values >54 mg/dL (<70 mg/dL). Due to the modality of CGM employed in the study (blinded recording with retrospective analysis), no specific interventions were undertaken in real time. 

The evaluation of hypoglycaemia in non-diabetic patients is complex—it can be associated with severe illness, malnutrition, medications, hormonal deficiencies, tumours, and GI surgery; in young children, innate errors in the metabolism should be taken into consideration [30].

Children with clinically significant hypoglycaemia subjected to our study were fed via PEG, one of whom received a lower than recommended caloric intake (continuous feeding) and one higher than recommended (bolus feeding); the percentage of time with alarm hypoglycaemia was 11 and 2.8%, respectively (Table 4). In both cases, during 81% of the period of CGM, glucose was within the normal range (TIR 70–140), which indicates that the probability of capturing severe hypoglycaemia using routine, one-time measurements of blood glucose (point of care) would be very low in the group of patients we studied. Meanwhile, the results of HbA1c evaluation (5/14 patients showed subnormal concentrations) suggest that hypoglycaemia may be a significant and underestimated issue in tube-fed patients with CNS impairment [31]. The assessment of HbA1c in children with CNS impairment fed artificially has not yet been studied by other authors. 

In our patients, the CGM was carried out in the inpatient setting, and the course of the feed supply was supervised by the parents/guardians accompanying the patients and medical staff at the same time. The actual consumption of the enteral diet was assessed, and the values obtained were compared to the recommended caloric intake calculated for each patient according to the Schofield formula based on growth age. Despite the optimisation of the patients’ nutritional therapy (the exclusion of factors that may interfere with feeding tolerance and the support of professional staff in the preparation and supply of the feed), it was still not possible to meet the estimated caloric requirements in more than half of the subjects (11/19; 57.89%; Table 2). We found that the actual energy intake fulfilled the amount estimated by the Schofield formula in only 2/19 children, and in 6/19, the intake was higher than recommended. Although in children fed with the bolus method the number of calories consumed per day was higher than in children fed with the continuous feeding method, no significant differences were found in the glycaemic profiles.

We have demonstrated a positive correlation between the mean daily glycaemia recorded using the CGM method and the patients’ BMI z-score (R = 0.48, *p* = 0.0397). The only statistically significant difference in the CGM parameters between the examined children, depending on their BMI, concerned the overnight time spent with glucose above 140 mg/dL (TAR 140; Table 5). The maximum of overnight TAR 140 in children with BMI ≥ 3 amounted to 1.6%, whereas none of the undernourished patients (BMI < 3 pc) presented with hyperglycaemia. The clinical relevance of this finding needs to be evaluated further, as the median values of overnight TAR 140 in both groups were at similar levels—0%. The percentages of time with glycaemic concentrations <70 mg/dL in children with BMI < 3 pc and BMI ≥ 3 pc were comparable (*p* = 0.2629).

Our results show that in the real-life setting, even with an optimised feeding modality (continuous delivery in bolus-intolerant children) and the modified energy density of the diet (6/19 children received a hypercaloric diet), in some patients, it is not possible to achieve an adequate energy supply. This limitation is due to the secondary, severe gastrointestinal dysfunction that characterises patients with severe CNS impairment—gross motor dysfunction leads to muscle contractures and musculoskeletal deformities, including, in particular, multidimensional spinal deformities, resulting in the displacement of gastrointestinal organs and the impairment of gastrointestinal motility. These disorders may manifest as gastro-oesophageal reflux, gastric-emptying disorders, and constipation, most often comorbid. The prevalence rate of GORD in patients with cerebral palsy is estimated to be 75%, and the prevalence rate of constipation was estimated to be between 26 and 74% [32]; according to Hollung et al. [33], as many as 95% of patients with cerebral palsy are affected by at least one comorbidity. These complications make tube feeding very difficult in patients with severe CNS impairment, and total enteral nutrition does not always effectively fulfil caloric requirements, so energy reserves may be too low to effectively maintain normoglycaemia. 

Another mechanism responsible for the occurrence of hypoglycaemia in tube-fed children with CNS impairments may be dumping syndrome. Dumping syndrome is a consequence of a rapid delivery of nutrients to the duodenum, which triggers a systemic neural and hormonal response. Based on International consensus on the diagnosis and management of dumping syndrome [34], the syndrome is subdivided into early (hyperglycaemia, gastrointestinal, and vasomotor symptoms occurring within the first hour after a meal) and late (reactive hypoglycaemia 1–3 h after a meal) forms. This pathology is a frequent complication of oesophageal and gastric surgery and was also reported in children after gastrostomy placement [35]. Dumping syndrome which occurs in non-surgical patients is classified as idiopathic. Diagnostic tests for dumping syndrome include: spontaneous plasma concentrations of glucose < 50 mg/dL and the modified oral glucose tolerance test (OGTT) as the preferred method. The suitability of CGM has not been verified. 

In addition, severe malnutrition and/or CNS damage alone may impair the mechanisms of the neuro-hormonal counter-regulation of glucose homeostasis, leading to hypoglycaemia [30,36,37,38]. Physiologically, under conditions of hypoglycaemia and low energy availability, brain anorexigenic glucose-inhibited neurons are activated, and consequently, glucagon (pancreas), adrenaline (adrenal medulla), ACTH (pituitary gland), and glucocorticoids (adrenal cortex) are released. Data regarding the HPA axis in undernourished children with severe CNS damage and utilising tube feeding are lacking; in patients with cerebral palsy, higher levels of cortisol compared with controls were reported [39]. 

It is also important to note that technical problems, such as incidentally pressing on the sensor, may lead to false-positive hypoglycaemia readings (this was excluded in our patients).

The results of our study suggest a need for the closer monitoring of glycaemic homeostasis and indicate that hypoglycaemia may be a genuine concern in children with CNS impairment utilising enteral nutrition. This is an important observation, as currently-published studies regarding the metabolic complications associated with tube feeding in non-diabetic patients have focused on subjects in critical conditions, under stress, after surgery, and often receiving glucocorticoids or vasoconstricting drugs [40,41,42,43,44]. In our study, we did not identify any patients meeting the criteria for a diagnosis of diabetes, further confirmed by normal glycated haemoglobin (HbA1C) values, which did not exceed the normal range in any of our patients. 

The main limitations of our study are the small group size, due in part to the inpatient observation model adopted in the protocol, and secondly, the relatively short duration of CGM. On the one hand, this ensured the optimisation of nutritional therapy and the accuracy of glycaemic monitoring but was nevertheless inconvenient for the patients’ parents/caregivers. 

We suggest that in children with severe CNS impairment, the implementation of the CGM system would be the most important in the initial stage of nutritional support, when the optimal composition, volume, and mode of delivery of the enteral diet are determined. In patients presenting with clinically relevant glucose alterations, extended clinical evaluation with the use of the latest real-time glucose monitoring systems with a hypoglycaemia/hyperglycaemia alarm [45] (in our current study, the blinded CGM system was utilised), alongside the detailed analysis of individual energy expenditure, body composition, nutritional support course, and endocrine factors should be considered.

## 5. Conclusions

Enteral nutrition delivered via nasogastric, nasoenteral, or percutaneous tubes in children with chronic CNS impairment is associated with the risk of hypoglycaemia, and CGM might be a useful tool to estimate the metabolic adequacy of enteral nutritional support in terms of glucose control.

## Figures and Tables

**Table 1:** The nutritional status and nutritional support characteristics of the study group (n = 19).

Variable		Description
Body weight (kg)—median (25–75 pc)		19.5 (15.7–25.5)
Body weight z-score—median (25–75 pc)		−4.83 (−7.95–−2.65)
Body length (m)—median (25–75 pc)		1.16 (1.12–1.4)
Body length z-score—median (25–75 pc)		−3.45 (−4.2–−2.24)
Growth age (years)—median (25–75 pc)		5 (4.5–9)
BMI z-score—median (25–75 pc)		−2.26 (−4.41–−1.07)
Intragastric feeding (n/%)		17/89.5
	PEG (n/%)	16/84.2
	NG (n/%)	1/5.3
Postpyloric feeding (n/%)		2/10.5
	PEG-PEJ (n/%)	2/10.5
BF (n/%)		13/68.4
CF (n/%)		6/31.6
100% SCF (n/%)		13/68.4
MD (SCF + HD) (n/%)		6/31.6

PEG—percutaneous endoscopic gastrostomy; NG—nasogastric tube; PEG-PEJ—percutaneous endoscopic gastro-jejunostomy; BF—bolus feeding; CF—continuous feeding; SCF—standardised commercially manufactured formula; MD—mixed diet = SCF covering at least 50% of demand + HD—blenderised home-prepared diet.

**Table 2:** Recommended and actual caloric intake of patients in this study (n = 19).

Variable	Description
ACI (kcal)—median (25–75 pc)	1000 (800–1000)
ACI/kg BW (kcal)—median (25–75 pc)	50 (37.7–68.8)
RCI (kcal)—median (25–75 pc)	1038.28 (954.98–1168.18)
RCI/kg BW (kcal)—median (25–75 pc)	50.09 (47.1–60.0)
ACI < RCI (n/%)	11/57.89
ACI > RCI (n/%)	6/31.58
ACI = RCI (n/%)	2/10.52

ACI—actual caloric intake; RCI—recommended caloric intake; BW—body weight.

**Table 3:** Continuous glucose monitoring results (n = 19).

Variable	Description
Measurement duration (hours)—mean ± SD/minimum	83 (66–101)/36
Number of recorded measurements in 1 patient—median (25–75 pc)	1001 (798–1221)
Mean glucose (mg/dL)—median (25–75 pc; CV)	97 (92–101; 14.3%)
Minimum recorded glucose level (mg/dL)	41
Maximum recorded glucose level (mg/dL)	180
TIR 70–140 (%)—median (25–75 pc)	98.06 (94.86–99.03)
TAR 140 (%)—median (25–75 pc)	0.74 (0–1.4)
TUR 70 (%)—median (25–75 pc)	1.32 (0.39–3.03)
Number of patients with glucose level < 70 mg/dL (n/%)	15/78.9
Number of patients with glucose level < 54 mg/dL (n/%)	2/10.53

CV—coefficient of variation; TIR 70–140—time in range of 70–140 mg/dL; TAR 140—time above range (glucose >140 mg/dL); TUR 70—time under range (glucose <70 mg/dL).

**Table 4:** The characteristics of patients with severe (<54 mg/dL) hypoglycaemia.

Variable	Patient 1	Patient 2
Age (years)	14.4	17.2
Sex	F	M
Clinical diagnosis	Cerebral palsy	Cerebral palsy
BMI z-score	−5.25	−3.4
Mode of enteral access/ modality of EF	PEG/ CF	PEG/ CF
Diet type	100% CD	100% CD
ACI kcal/kg BW	37.2	55
RCI kcal/kg BW	48.6	40.1
Number of episodes of hypoglycaemia <54 mg/dL	1	3
TIR 70–140 (%)	81.3	81.06
TUR 70 (%)	18	18
TUR 54 (%)	11	2.74

F—female; M—male; EF—enteral feeding; PEG—percutaneous endoscopic gastrostomy; CF—continuous feeding; CD—standardised commercially manufactured diet; ACI—actual caloric intake; RCI—recommended caloric intake; BW—body weight; TIR 70–140—time in range of 70–140 mg/dL; TUR 70—time under range of <70 mg/dL; TUR 54—time under range of <54 mg/dL.

**Table 5:** Characteristics of patients in study with reference to BMI-pc (n = 19).

Variable	BMI < 3 pc n = 11	BMI ≥ 3 pc n = 8	*p*
Age (years)	12.43 (6.17–16.42)	9.76 (5.46–15.07)	0.4328
Clinical diagnosis (n/%)	CP (8/72.7) Other (3/27,3)	CP (5/62.5) Other (3/37.5)	1.0000
Diet composition (n/%)	CD (8/72.7) MD (3/27.3)	CD n = 7/87.5 MD n = 1/12.5	0.1770
Modality of EF (n/%)	BF (9/81.8) CF (2/18.2)	BF (4/50) CF (4/50)	0.3189
ACI kcal/kg BW	54.56 (40.80–68.80)	50.60 (28.15–73.20)	0.3859
TAR 140 overnight (%)	0 (0.00–0.0)	0.0 (0.00–1.6)	0.0375
TUR 70 Entire recording (%)	1.8 (0.5–14.4)	0.77 (0.13–2.2)	0.2629

CP—cerebral palsy; CD—standardised commercially manufactured diet; MD—mixed diet = CD covering at least 50% of demand + HD—blenderised home-prepared diet; EF—enteral feeding; BF—bolus feeding; CF—continuous feeding; ACI—actual caloric intake; BW—body weight; TAR 140—time above range (glucose > 140 mg%); TUR 70—time under range (glucose < 70 mg%).

## Data Availability

Requests for access to data, may be made by contacting the corresponding author.

## References

[1] Romano C., van Wynckel M., Hulst J., Broekaert I., Bronsky J., Dall’Oglio L., Mis N.F., Hojsak I., Orel R., Papadopoulou A. (2017). European Society for Paediatric Gastroenterology, Hepatology and Nutrition guidelines for the evaluation and treatment of gastrointestinal and nutritional complications in children with neurological impairment. J. Pediatr. Gastroenterol. Nutr..

[2] Romano C., Dipasquale V., Gottrand F., Sullivan P.B. (2018). Gastrointestinal and nutritional issues in children with neurological disability. Dev. Med. Child Neurol..

[3] Becker P., Carney L.N., Corkins M., Monczka J., Smith E., Smith S.E., Spear B.A., White J.V. (2015). Consensus Statement of the Academy of Nutrition and Dietetics/American Society for Parenteral and Enteral Nutrition: Indicators Recommended for the Identification and Documentation of Pediatric Malnutrition (Undernutrition). Nutr. Clin. Pract..

[4] Andrew M.J., Parr J.R., Sullivan P.B. (2012). Feeding difficulties in children with cerebral palsy. Arch. Dis. Child. Educ. Pract. Ed..

[5] Trivić I., Hojsak I. (2019). Evaluation and Treatment of Malnutrition and Associated Gastrointestinal Complications in Children with Cerebral Palsy. Pediatr. Gastroenterol. Hepatol. Nutr..

[6] DiMeglioa L.A., Acerinib C.L., Codnerc E., Craigd M.E., Hofere S.E., Pillayf K., Maahsg D.M. (2018). Glycemic control targets and glucose monitoring for children, adolescents, and young adults with diabetes, 2018 ISPAD Clinical Practice Consensus Guidelines. Pediatric Diabetes.

[7] Littler H., Tume L.N. (2022). Is bolus or continuous enteral feeding better in critically ill children: An evidence-based review. Nurs. Crit. Care.

[8] Palisano R., Rosenbaum P., Bartlett D., Livingston M.H. (2008). Content validity of the expanded and revised Gross Motor Function Classification System. Dev. Med. Child. Neurol..

[9] Roostaei M., Baharlouei H., Azadi H., Fragala-Pinkham M.A. (2017). Effects of Aquatic Intervention on Gross Motor Skills in Children with Cerebral Palsy: A Systematic Review. Phys. Occup. Ther. Pediatr..

[10] Rempel G. (2015). The Importance of Good Nutrition in Children with Cerebral Palsy. Phys. Med. Rehabil. Clin. N. Am..

[11] Scarpato E., Staiano A., Molteni M., Terrone G., Mazzocchi A., Agostoni C. (2017). Nutritional assessment and intervention in children with cerebral palsy: A practical approach. Int. J. Food Sci. Nutr..

[12] World Health Organization Recommendations for Data Collection, Analysis and Reporting on Anthropometric Indicators in Children under 5 Years Old. https://www.who.int/nutrition/team/en/.

[13] Kułaga Z., Różdżyńska-Świątkowska A., Grajda A., Gurzkowska B., Wojtyło M., Góźdź M., Świąder-Leśniak A., Litwin M. (2015). Siatki centylowe dla oceny wzrastania i stanu odżywienia polskich dzieci i młodzieży od urodzenia do 18 roku życia, Stand. Med. Pediatr..

[14] Kuczmarski R., Ogden C., Guo S., Grummer-Strawn L.M., Flegal K., Mei Z., Wei R., Curtin L., Roche A., Johnson C. (2002). 2000 CDC Growth Charts for the United States: Methods and Development. National Center for Health Statistics. Vital Health Stat..

[15] Fuentes-Servín J., Avila-Nava A., González-Salazar L.E., Pérez-González O.A., Servín-Rodas M.D.C., Serralde-Zuñiga A.E., Medina-Vera I., Guevara-Cruz M. (2021). Resting Energy Expenditure Prediction Equations in the Pediatric Population: A Systematic Review. Front. Pediatr..

[16] Czerwoniuk D., Fendler W., Walenciak L., Mlynarski W. (2011). GlyCulator: A Glycemic Variability Calculation Tool for Continuous Glucose Monitoring Data. J. Diabetes. Sci. Technol..

[17] International Hypoglycaemia Study Group (2017). Glucose concentrations of less than 3.0 mmol/l (54 mg/dL) should be reported in clinical trials: A joint position statement of the American Diabetes Association and the Europian Association for the Study of Diabetes. Diabetologia.

[18] Araszkiewicz A., Bandurska-Stankiewicz E., Borys S., Budzyński A., Cyganek K., Cypryk K., Czech A., Czupryniak L., Drzewoski J., Dzida G. (2021). 2021 Guidelines on the management of patients with diabetes. A position of Diabetes Poland. Clin. Diabetol..

[19] Dahl M., Thommessen M., Rasmussen M., Selberg T. (1996). Feeding and nutritional characteristics in children with moderate or severe cerebral palsy. Acta Pediatr..

[20] Wyszomirska K., Wyszomirski A., Brzezinski M., Borkowska A., Zagierski M., Kierkus J., Ksiazyk J., Romanowska H., Swider M., Toporowska-Kowalska E. (2021). Home Artificial Nutrition in Polish Children: An Analysis of 9-Year National Healthcare Provider Data. Nutrients.

[21] Lezo A., Capriati T., Spagnuolo M.I., Lacitignola L., Goreva I., Di Leo G., Cecchi N., Gandullia P., Amarri S., Forchielli M.L. (2018). Paediatric Home Artificial Nutrition in Italy: Report from 2016 Survey on Behalf of Artificial Nutrition Network of Italian Society for Gastroenterology, Hepatology and Nutrition (SIGENP). Nutrients.

[22] Marchand V., Motil K.J., NASPGHAN Committee on Nutrition (2006). Nutrition support for neurologically impaired children: A clinical report of the North American Society for Pediatric Gastroenterology, Hepatology, and Nutrition. J. Pediatr. Gastroenterol. Nutr..

[23] Calis E.A., Veugelers R., Sheppard J.J., Tibboel D., Evenhuis H.M., Penning C. (2008). Dysphagia in children with severe generalized cerebral palsy and intellectual disability. Dev. Med. Child. Neurol..

[24] Coad J., Toft A., Lapwood S., Manning J., Hunter M., Jenkins H., Sadlier C., Hammonds J., Kennedy A., Murch S. (2017). Blended foods for tube-fed children: A safe and realistic option? A rapid review of the evidence. Arch. Dis. Child..

[25] Dipasquale V., Diamanti A., Trovato C.M., Elia D., Romano C. (2022). Real food in enteral nutrition for chronically ill children: Overview and practical clinical cases. Curr. Med. Res. Opin..

[26] Battelino T., Danne T., Bergenstal R.M., Amiel S.A., Beck R., Biester T., Bosi E., Buckingham B.A., Cefalu W.T., Close K.L. (2019). Clinical targets for continuous glucose monitoring data interpretation: Recommendations from the International Consensus on Time in Range. Diabetes Care.

[27] Klonoff D.C., Nguyen K.T., Xu N.Y., Gutierrez A., Espinoza J.C., Vidmar A.P. (2022). Use of Continuous Glucose Monitors by People Without Diabetes: An Idea Whose Time Has Come?. J. Diabetes Sci. Technol..

[28] Shah V.N., DuBose S.N., Li Z., Beck R.W., Peters A.L., Weinstock R.S., Kruger D., Tansey M., Sparling D., Woerner S. (2019). Continuous Glucose Monitoring Profiles in Healthy Nondiabetic Participants: A Multicenter Prospective Study. J. Clin. Endocrinol. Metab..

[29] DuBose S.N., Kanapka L.G., Bradfield B., Sooy M., Beck R.W., Steck A.K. (2022). Continuous Glucose Monitoring Profiles in Healthy, Nondiabetic Young Children. J. Endocr. Soc..

[30] Casertano A., Rossi A., Fecarotta S., Rosanio F.M., Moracas C., Di Candia F., Parenti G., Franzese A., Mozzillo E. (2021). An Overview of Hypoglycaemia in Children Including a Comprehensive Practical Diagnostic Flowchart for Clinical Use. Front. Endocrinol..

[31] Uyar S., Gorar S., Kok M., Ozer H., Koker G., Bostan F., Cekin A.H. (2018). Could Insulin and Hemoglobin A1c Levels be Predictors of Hunger- Related Malnutrition/Undernutrition Without Disease?. Clin. Lab..

[32] Penagini F., Mameli C., Fabiano V., Brunetti D., Dilillo D., Zuccotti G.V. (2015). Dietary Intakes and Nutritional Issues in Neurologically Impaired Children. Nutrients.

[33] Hollung S., Bakken I., Vik T., Lydersen S., Wiik R., Aaberg K.M., Andersen G.L. (2019). Comorbidities in cerebral palsy: A patient registry study. Dev. Med. Child. Neuro..

[34] Scarpellini E., Arts J., Karamanolis G., Laurenius A., Siquini W., Suzuki H., Ukleja A., Van Beek A., Vanuytsel T., Bor S. (2020). International consensus on the diagnosis and management of dumping syndrome. Nat. Rev. Endocrinol..

[35] Di Leo G., Pascolo P., Hamadeh K., Trombetta A., Ghirardo S., Schleef J., Barbi E., Codrich D. (2019). Gastrostomy Placement and Management in Children: A Single-Center Experience. Nutrients.

[36] López-Gambero A.J., Martínez F., Salazar K., Cifuentes M., Nualart F. (2019). Brain Glucose-Sensing Mechanism and Energy Homeostasis. Mol Neurobiol..

[37] Bansal N., Weinstock R.S., Feingold K.R., Anawalt B., Boyce A., Chrousos G., de Herder W.W., Dhatariya K., Dungan K., Hershman J.M., Hofland J., Kalra S. (2000). Non-Diabetic Hypoglycaemia. Endotext [Internet].

[38] Martin-Grace J., Dineen R., Sherlock M., Thompson C.J. (2020). Adrenal insufficiency: Physiology, clinical presentation and diagnostic challenges. Clin Chim Acta.

[39] Malaeb S.N., Stonestreet B.S. (2014). Steroids and injury to the developing brain: Net harm or net benefit?. Clin. Perinatol..

[40] Valizadeth Hasanloei M.A., Vahdat Shariatpanahi Z., Davoud Vahabzadeh D., Vahabzadeh D., Nasiri L., Shargh A. (2017). Non-diabetic Hyperglycemia and Some of Its Correlates in ICU Hospitalized Patients Receiving Enteral Nutrition. J. Clin. Med..

[41] Gosmanov A.R., Umpierrez G.E. (2013). Management of Hyperglycemia During Enteral and Parenteral Nutrition Therapy. Curr. Diab. Rep..

[42] Davidson P., Kwiatkowski C.A., Wien M. (2015). Management of Hyperglycemia and Enteral Nutrition in the Hospitalized Patient. Nutr. Clin. Pract..

[43] Shahriari M., Rezaei E., Bakht L.A., Abbasi S. (2015). Comparison of the effects of enteral feeding through the bolus and continuous methods on blood sugar and prealbumin levels in ICU inpatients. J. Educ. Health Promot..

[44] Chang-Jie R., Yao B., Tuo M., Lin H., Wan X.-Y., Pang X.-F. (2021). Comparison of sequential feeding and continuous feeding on the blood glucose of critically ill patients: A non-inferiority randomized controlled trial. Chin. Med. J..

[45] Cappon G., Vettoretti M., Sparacino G., Facchinetti A. (2019). Continuous Glucose Monitoring Sensors for Diabetes Management: A Review of Technologies and Applications. Diabetes Metab. J..

